# Notes

**Published:** 1898-09-10

**Authors:** 


### The Institutional Workshop.

### NOTES.

The site for the new Royal Infirmary at Newcastle-on-Tyne has been sanctioned by Act of Parliament.

Mrs. Beauchamp last week opened a new workhouse infirmary at Redruth, for the use of the Union.

The Thompson-Yates Laboratories of Physiology and Pathology are to be opened on October 8th by Lord Lister, who will receive an honorary degree from the Victoria University and be entertained at a banquet by the Mayor of Liverpool.

At the last meeting of the Castlereagh Dispensary Committee, some very unsatisfactory statements as to the sanitation of many dwellings in Belfast were made. In one case, where his patient had died of typhoid fever, the doctor in attendance reported that he had discovered a sewer below the bedroom window with no covering over the trap. Another member of the committee stated that some 21 houses in a particular street had no gulley-trap in use, and that the smells were most offensive.

Gerardmer, the little place in the Vosges Mountains, to which it is stated that the Marquis of Salisbury will go with his family after leaving Contrexeville, stands about 2,375 ft. above the sea-level. It has a hydropathic establishment, which is open from May to October 1st, and contains good baths and complete arrangements for carrying out hydropathic treatment, together with pine baths and other accessories. A new establishment was opened in 1892. It is not very far from Contrexeville, which, indeed, is situated in the same mountain range, but much lower down.

The report of the Inspector of Lunatic Asylums in Victoria, just issued, is not one revealing great progress. There is a lack of accommodation, taken as a whole, and overcrowding in some instances. The boarding-out system has not expanded, and shows no prospect of extension. Better classification in some cases is advised, and the unsatisfactory result of the constant change in the staff of attendants is commented on and considered due to inadequate payment. The weekly rate of expenditure per patient has increased from 8s. 3d. to 8s. 6d. per week. Provisions have cost more, and now the diet-scale for attendants is to be improved. The ratio of admissions to the asylums to the sane population in Victoria is one to every 271.

Last week the Duke of Devonshire laid the foundation-stone of a new hospital at Keighley. The building consists of four blocks. One of these is for the administration, the others contain the wards. Two of these are to be 70 ft. by 27 ft., each containing 16 beds. There are also two four-bed wards, and four single rooms. Two day-rooms are provided, and the necessary offices. At the south end of each of the large wards there is to be a verandah. The operating block is situated between the above pavilions, and at the back of the buildings the mortuary will be situated. The cost of the hospital will be upwards of £20,000, and it is hoped to raise an endowment fund of £10,000 also.

